# Nurse-led non-pharmacological interventions for cognitive improvement in dementia patients: a systematic review and meta-analysis of randomized controlled trials

**DOI:** 10.3389/fnagi.2026.1876966

**Published:** 2026-07-24

**Authors:** Jie Zhang, Rong Luo, Jialei Chen

**Affiliations:** 1 General Practice Ward/International Medical Center Ward, General Practice Medical Center, West China Hospital, Sichuan University, Chengdu, China; 2 Department of Orthopedics Surgery, Trauma Medical Center, Orthopedic Research Institute, West China Hospital, Sichuan University, Chengdu, China

**Keywords:** cognitive function, dementia, meta-analysis, non-pharmacological interventions, nurse-led

## Abstract

**Introduction:**

Nurse-led non-pharmacological interventions (NLNPIs) have gradually been recognized as a safe strategy to improve cognitive function in patients with dementia. Nevertheless, current research findings remain controversial and the adequacy of sample sizes in previous investigations has not been clarified. This study aimed to evaluate the efficacy of NLNPIs in improving cognitive function in patients with dementia.

**Methods:**

A systematic literature search for eligible English publications was conducted across three electronic databases (PubMed, EMBASE, and Scopus) on November 25, 2025. Narrative synthesis was adopted to summarize the characteristics of NLNPIs, while a meta-analysis was performed to quantitatively pool effect sizes regarding intervention efficacy.

**Results:**

A total of 18 randomized controlled trials (RCTs) involving 1,858 patients were included in the systematic review, among which 13 trials were eligible for quantitative meta-analysis. Pooled analyses revealed that NLNPIs significantly improved post-intervention cognitive function assessed immediately upon intervention termination, compared with routine nursing care. Moreover, a cumulative intervention duration (CID) exceeding 480 min was identified as a potential threshold for achieving meaningful cognitive benefits. In terms of long-term efficacy, no statistically significant cognitive improvements were observed at the 6-month follow-up after intervention completion.

**Conclusions:**

NLNPIs effectively improve cognitive function in patients with dementia, with therapeutic benefits sustained at least until the end of the intervention. Furthermore, the intervention effect may follow a dose-dependent pattern. Large-sample, high-quality RCTs with standardized intervention protocols and rigorous blinding designs are warranted to further validate long-term outcomes and optimal dose thresholds, thereby generating more robust evidence for clinical dementia care.

**Systematic review registration:**

https://www.crd.york.ac.uk/PROSPERO/view/CRD420251228955, identifier: CRD420251228955.

## 1 Introduction

Dementia is an irreversible neurodegenerative disorder characterized by cognitive dysfunction, depressive symptoms, behavioral and psychological disturbances, and impairments in activities of daily living (ADL). Globally, it ranks as the fifth leading cause of mortality and the most burdensome disease in terms of disability-adjusted life years (G. B. D., Neurology Collaborators, 2019).

The global prevalence of dementia is escalating rapidly, with projections estimating 82 million cases by 2030 and 152 million by 2050 (World Health Organization, 2019). Cholinesterase inhibitors are widely prescribed to alleviate cognitive symptoms in dementia patients; however, 20%−30% of those treated experience adverse effects, including gastrointestinal discomfort (abdominal pain, nausea, vomiting) and anorexia. These limitations highlight the need for non-pharmacological interventions (NPIs), which demonstrate superior safety profiles and efficacy in managing cognitive decline, ADL dysfunction, and neuropsychiatric symptoms across dementia subtypes (Saragih et al., 2021).

The World Alzheimer Report 2022 underscores that diagnosis represents merely the first step in the dementia care continuum (Gauthier et al., 2022). Integrated treatment, care, and support constitute the cornerstone of effective post-diagnostic models, with early-stage interventions—implemented with precision and adherence to evidence-based protocols—yielding substantial economic benefits (Chao et al., 2020). Nurses frequently serve as the primary contact in post-diagnosis care planning, treatment delivery, and ongoing monitoring, playing a pivotal role in supporting individuals with Alzheimer's disease (AD) and their caregivers.

Consequently, nurses are the preferred healthcare professionals for delivering health promotion and patient-centered care. Nursing-led interventions have demonstrated efficacy in reducing diagnostic delays, optimizing resource utilization, lowering costs, and enhancing patient safety and satisfaction. Nurses act as core providers of continuous, person-centered dementia care across nursing homes, community centers and home-based settings. Equipped with standardized hands-on training in dementia non-pharmacological therapies and maintaining long-term regular contact with patients and family caregivers, nurses possess unique strengths in delivering individualized non-pharmacological interventions. They are able to dynamically adjust intervention regimens, provide health education for caregivers simultaneously, and implement therapies across diverse care environments, making them the most suitable professionals to deliver interventions targeting cognitive improvement in people living with dementia (Bergman et al., 2013; Chao et al., 2020; Gauthier et al., 2022).

However, current evidence on NLNPIs for dementia remains inconsistent. A systematic review analyzed 24 studies, of which 15 were included in a meta-analysis comprising 8 RCTs and 7 quasi-experimental studies. This analysis revealed that NLNPIs significantly improved cognitive function in dementia patients (Suh et al., 2023). Conversely, another meta-analysis reported contrasting findings, suggesting that nursing interventions primarily alleviate depressive symptoms and enhance quality of life, with limited impact on cognitive outcomes (Huang et al., 2023).

Existing studies concerning NLNPIs only report binary outcomes of effective or ineffective treatment. Nevertheless, identical intervention modalities yield vastly different therapeutic effects across varying intervention durations and frequencies, leaving frontline clinical nurses unable to confirm standardized intervention thresholds. Moreover, no prior systematic review has quantitatively integrated dose parameters to characterize the dose-response association with treatment efficacy, resulting in a lack of unified practical reference for clinical implementation.

To resolve inconsistencies and deficiencies in the conclusions of available studies, the present study conducts a systematic review and meta-analysis of relevant literature published since 2000. It comprehensively evaluates the cognitive benefits of NLNPIs for patients with dementia and further performs an exploratory analysis of the underlying dose-response relationship.

Compared with previous systematic reviews in this field, the present study has three core innovations. First, we conducted an exploratory dose-response analysis of intervention efficacy, a component absent from all prior relevant reviews. Existing syntheses merely qualitatively summarized the overall therapeutic effects of nursing interventions without quantifying correlations between CID and clinical benefits; our analysis generates quantitative evidence for identifying intervention thresholds and optimal dosage ranges. Second, this review adopted stricter inclusion criteria limited exclusively to RCTs, yielding evidence of superior quality. Third, we extended the literature retrieval timeframe and incorporated newly published high-quality RCTs to update the field's evidence base, expand the overall sample size, and enhance the timeliness and comprehensiveness of our conclusions.

## 2 Methods

### 2.1 Study design

A systematic review incorporating narrative synthesis and meta-analysis was conducted to synthesize data from the included studies, ensuring a rigorous and comprehensive evaluation of the evidence. The protocol has been registered to PROSPERO (CRD 420251228955).

### 2.2 Search methods

A structured, systematic literature search was performed across three electronic databases: PubMed, EMBASE, and Scopus. Supplementary hand-searching was conducted to identify additional relevant studies. Table 1 details the search strategy and retrieval outcomes for each database. Boolean operators (“AND”/“OR”) were employed to combine key search terms, ensuring precision and inclusivity. Microsoft Excel and EndNote were utilized for efficient management of the search process and documentation of findings.

**Table 1:** Search strategy in electronic databases.

Database	Search strategy	Results
PubMed	((“dementia”[MeSH Terms] OR “dementia”[All Fields] OR “dementias”[All Fields] OR “dementia s”[All Fields] OR (“alzheime s”[All Fields] OR “alzheimer disease”[MeSH Terms] OR (“alzheimer”[All Fields] AND “disease”[All Fields]) OR “alzheimer disease”[All Fields] OR “alzheimer”[All Fields] OR “alzheimer s”[All Fields] OR “alzheimers”[All Fields] OR “alzheimers s”[All Fields])) AND (“cognition”[MeSH Terms] OR “cognition”[All Fields] OR “cognitions”[All Fields] OR “cognitive”[All Fields] OR “cognitively”[All Fields] OR “cognitives”[All Fields] OR (“cognition”[MeSH Terms] OR “cognition”[All Fields] OR “cognitions”[All Fields] OR “cognitive”[All Fields] OR “cognitively”[All Fields] OR “cognitives”[All Fields]) OR (“cognition”[MeSH Terms] OR “cognition”[All Fields] OR (“cognitive”[All Fields] AND “function”[All Fields]) OR “cognitive function”[All Fields])) AND (“nurse-led”[All Fields] OR (“nurse s”[All Fields] OR “nurses”[MeSH Terms] OR “nurses”[All Fields] OR “nurse”[All Fields] OR “nurses s”[All Fields])) AND (“non-pharmacologic”[All Fields] OR “non-pharmacological”[All Fields] OR (“therapeutics”[MeSH Terms] OR “therapeutics”[All Fields] OR “treatments”[All Fields] OR “therapy”[MeSH Subheading] OR “therapy”[All Fields] OR “treatment”[All Fields] OR “treatment s”[All Fields]) OR (“therapeutics”[MeSH Terms] OR “therapeutics”[All Fields] OR “therapies”[All Fields] OR “therapy”[MeSH Subheading] OR “therapy”[All Fields] OR “therapy s”[All Fields] OR “therapys”[All Fields]) OR (“intervention s”[All Fields] OR “interventions”[All Fields] OR “interventive”[All Fields] OR “methods”[MeSH Terms] OR “methods”[All Fields] OR “intervention”[All Fields] OR “interventional”[All Fields]))) AND ((english[Filter]) AND (2000:2025[pdat]))	1009
EMBASE	((dementia OR alzheimer) AND (cognition OR cognitive function OR cognitive) AND (nurse-led OR nurse) AND (non-pharmacologic OR treatment OR treatment OR intervention)).mp. [mp = title, abstract, heading word, drug trade name, original title, device manufacturer, drug manufacturer, device trade name, keyword heading word, floating subheading word, and candidate term word] and (english language and yr =“2000–2025”)	610
SCOPUS	(ALL (dementia OR alzheimer) AND ALL (cognition OR “cognitive function” OR cognitive) AND ALL (“nurse-led” OR nurse) AND ALL (non-pharmacologic OR treatment OR intervention) AND LANGUAGE (english) AND PUBYEAR > 1999 AND PUBYEAR < 2026)	711
Total		2330

### 2.3 Inclusion and exclusion criteria

This review evaluated the evidence reported in studies identified using the PICOT framework as follows: (1) Population: Participants living with dementia with no limitations on age, gender, types of dementia, severity of dementia, or care setting. (2) Intervention: NLNPIs to improve cognition in people with dementia (i.e., music therapy, reminiscence therapy (RT), cognitive therapy, massage, mindfulness therapy, and so on). (3) Comparator: Usual care or standard nursing for people with dementia. (4) Outcome: cognitive function. (5) Time: The publication dates were limited from 2000 to February, 2025. Only RCTs were included. The language of published studies was restricted to English. But the following types of study were excluded: (1) animal studies; (2) studies not published in English; (3) studies in which the relevant data could not be extracted, and the original author was contacted without response; and (4) review articles, expert opinions, case reports, and letters to editors.

### 2.4 Quality appraisal

The RCTs were evaluated using the Cochrane Collaboration's tool for assessing the risk of bias, which includes the following aspects: (1) random-sequence generation (selection bias); (2) allocation concealment (selection bias); (3) blinding of participants and personnel (performance bias); (4) blinding of outcome assessment (detection bias); (5) incomplete outcome data (attrition bias); (6) selective reporting (reporting bias); and (7) other bias. Two independent reviewers conducted a quality assessment and resolved differences through discussion with a third reviewer.

### 2.5 Data extraction

Two reviewers independently extracted the data from all the included studies using a standardized data extraction form to ensure uniform collection. The eligible full-text articles needed to have sufficient data to extract and pool. If the relevant data were not provided in the article, the authors were contacted via email to request the data. The following data were extracted from all eligible studies. (1) Study characteristics: first author, publication year, country, setting, sample size of different groups, patient demographics (age, gender), type of dementia, stage of dementia, and cognitive measurement; (2) Intervention characteristics: intervention provider, type of cognitive intervention, contents of intervention, length of single individual intervention session, number of sessions per week, total weeks of intervention, time points for data collection, and main outcomes. A third investigator resolved any disagreements through discussion or verification.

### 2.6 Statistical analysis

The results of the studies were analyzed using RevMan 5.3 (The Nordic Cochrane Center, Copenhagen, The Cochrane Collaboration, 2014). If the data were sufficiently homogeneous (clinically and statistically), we summarized them in a meta-analysis. Continuous outcomes were calculated and expressed as the weighted mean difference with a 95% confidence interval (CI). To measure heterogeneity between studies, we used the χ2 test (a *p* value less than 0.10 indicates heterogeneity) and the *I*^2^ statistic (a value of less than 50% represents low heterogeneity and a value of 75% or more indicates high heterogeneity). A fixed-effects model was used in the meta-analysis, however, we used the random-effects model when significant heterogeneity among the studies was found. Forest plots were used to graphically represent the difference in outcomes between groups and for all included studies. If *P* was < 0.05, the results were considered statistically significant. Sensitivity analysis was performed to investigate the source of heterogeneity and verify the reliability of the results by excluding low-quality studies. A funnel plot was examined to evaluate the likelihood of publication bias.

### 2.7 Subgroup analysis

The subgroup analysis evaluated the effects of NLNPIs based on a single criterion: intervention dose, defined as CID. The CID was calculated as the product of these three dimensions:

- Session duration (length of single individual intervention session),
- Frequency (number of sessions per week), and
- Duration of intervention (total weeks of intervention).

Accordingly, subgroups were categorized into low-dose and high-dose groups based on the data distribution derived from the continuous dose-response models of included studies. All potential segmentation points of cumulative intervention duration were traversed and tested via subgroup meta-analyses to identify an optimal cut-off value capable of distinguishing studies with meaningful cognitive improvements from those without detectable therapeutic effects. Intergroup differences in intervention efficacy were subsequently compared.

## 3 Results

### 3.1 Characteristics of the included studies

#### 3.1.1 Study characteristics

**Figure 1:**
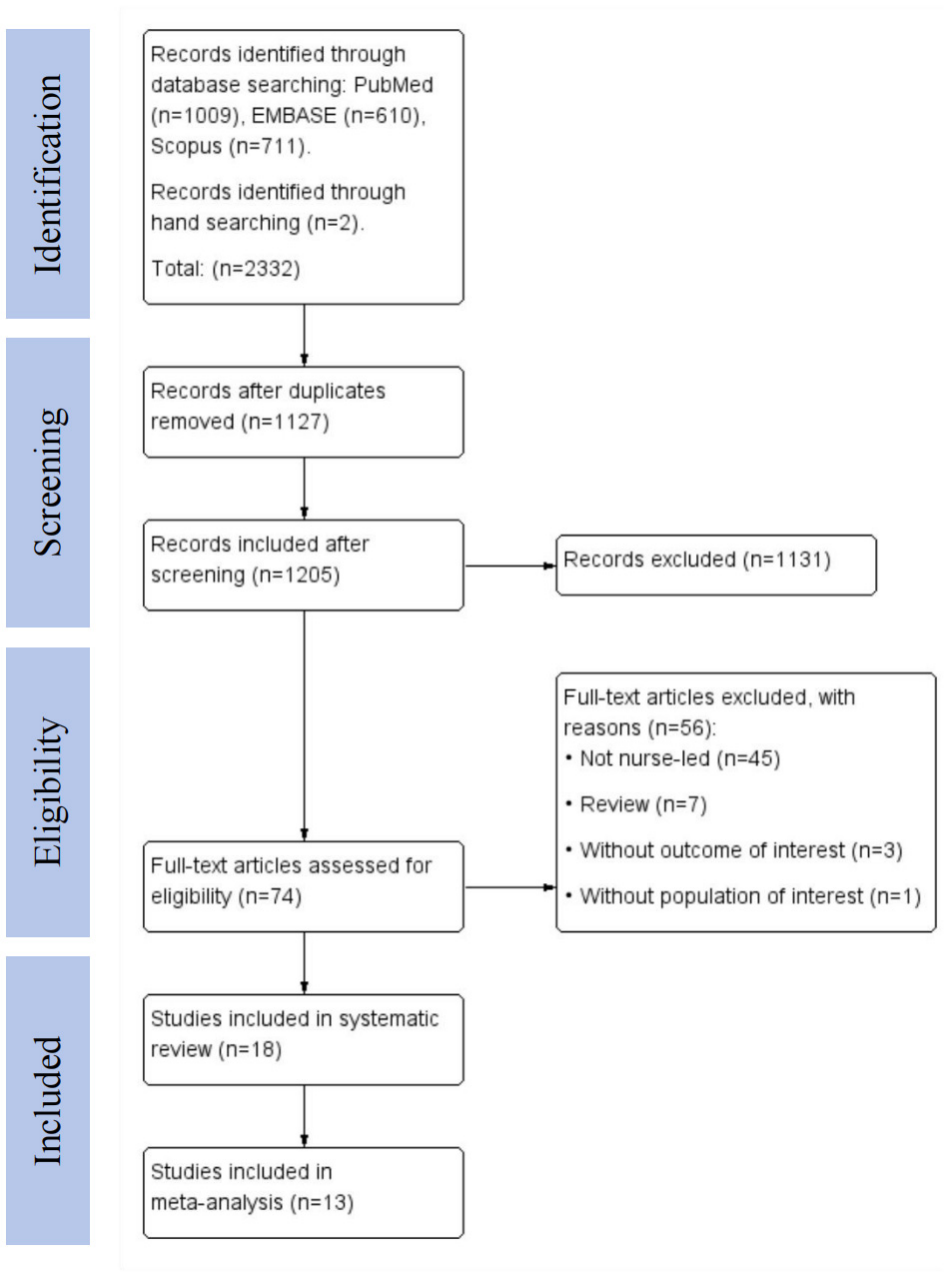
Flow diagram for the identification and selection of studies included in the meta-analysis.

Figure 1 presents the PRISMA flow diagram that mapped out the number of records identified, included, and excluded, and the reasons for exclusions in each step of this review (Additional file 1: PRISMA checklist). The key features of the 18 included studies are summarized in Table 2. These studies, published between 2004 and September 2024, enrolled 1,858 participants (1,021 in intervention groups and 837 in control groups), with sample sizes ranging from 29 to 407 and mean ages spanning 70.8 to 86.8 years. The majority of participants were diagnosed with dementia (including AD, vascular dementia, Lewy body dementia, primary degenerative dementia, and mixed dementia), mild cognitive impairment, or subjective cognitive decline.

**Table 2:** Characteristics of included studies.

No.	First author (year)	Country/setting	Participant characteristics						
			Group	Sample size	Mean age (SD)	Male/Female	Type of dementia	Stage of dementia	Cognitive measurement
1	Lai et al. (2004)	China/nursing home	IG	36	86.2 (6.3)	10/26	Dementia	DSM-IV	MMSE
			CG	30	86.8 (7.3)	11/19			
2	Callahan et al. (2006)	USA/care facility	IG	84	77.4 (5.9)	45/39	AD	DSM-III	MMSE
			CG	69	77.7 (5.7)	42/27			
3	Tadaka and Kanagawa (2007)	Japan/care facility	IG	AD 12 VD 18	AD 82.5 (6.6) VD 85.3 (6.3)	AD 4/8 VD 5/13	AD and VD	CDR=1-2	MMSE
			CG	AD 12 VD 18	AD 81.2 (6.2) VD 83.2 (6.4)	AD 2/10 VD 7/11			
4	Wang (2007)	China/care facility	IG	51	79.8 (6.3)	27/24	Dementia	CDR=1-3	MMSE
			CG	51	78.9 (7.6)	25/26			
5	Chien and Chan (2009)	China/community	IG	40	NR	24/16	AD	DSM-IV, mild or moderate stage	MMSE
			CG	40	NR	22/18			
6	Hsieh et al. (2010)	China/nursing home	IG	29	77.9 (5.6)	16/13	Dementia	CDR, mild or moderate stage	AES-C
			CG	32	77.25 (10.49)	20/12			
7	^*^ Graessel et al. (2011)	Germany/nursing home	IG	50	84.5 (4.5)	6/44	Primary degenerative dementia	MMSE < 24	ADAS-Cog
			CG	46	85.7 (5.7)	10/36			
8	^*^ Luttenberger et al. (2012)	Germany/nursing home	IG	30	84.1 (5.02)	3/27	Primary degenerative dementia	MMSE < 24	ADAS-Cog
			CG	22	84.64 (5.45)	6/16			
9	^*^ Waldorff et al. (2012)	Denmark/memory clinic	IG	163	76.5 (7.7)	76/87	AD, mixed AD with VD, Lewy body dementia	MMSE ≥ 20	MMSE
			CG	167	75.9 (6.6)	75/92			
10	^*^ Phung et al. (2013)	Denmark/memory clinic	IG	163	76.5 (7.7)	76/87	AD, mixed AD with VD, Lewy body dementia	MMSE ≥ 20	MMSE
			CG	167	75.9 (6.6)	75/92			
11	Van Bogaert et al. (2013)	Belgium/Care facility	IG	41	83 (NR)	4/37	AD	MMSE>18(mild) MMSE ≤ 18(moderate)	MMSE
			CG	41	85 (NR)	10/31			
12	Jha et al. (2013)	UK/community	IG	24	78.47 (8.0)	7/17	AD, VD, MCI, other dementia	MMSE>24(suspected) MMSE = 20–23(mild) MMSE < 20(moderate)	MMSE
			CG	24	79.00 (7.6)	9/15			
13	Thyrian et al. (2017)	Germary/home	IG	291	80.6 (5.7)	113/178	Dementia	DemTect produre	MMSE
			CG	116	79.8 (5.0)	46/70			
14	Bademli et al. (2019)	Türkiye/care facility	IG	30	NR	12/18	AD	MMSE = 13–24	MMSE
			CG	30	NR	14/16			
15	Lok et al. (2020)	Türkiye/neurology polyclinic	IG	30	NR	14/16	AD	MMSE = 13–24	MMSE
			CG	30	NR	16/14			
16	Yilmaz and Asiret (2021)	Türkiye/Nursing home	IG	15	85.53 (5.89)	5/10	Dementia	MMSE ≤ 17	MMSE
			CG	14	82.3 (7.55)	9/5			
17	Juniarti et al. (2021)	Indoniesia/community	IG	45	NR	10/35	Dementia	MMSE=19-26	MMSE
			CG	45	NR	15/30			
18	Yan et al. (2024)	China/memory clinic and community	IG	72	72.3 (6.2)	22/50	AD, MCI, subjective cognitive decline	MoCA	MoCA
			CG	72	70.8 (5.6)	28/44			

^*^Four studies independently drew on shared patient databases from Germany and Denmark for serial follow-up analyses.

AD, Alzheimer's Disease; ADAS-Cog, Alzheimer's Disease Assessment Scale-Cognitive Subscale; AES-C, Alzheimer's Disease Evaluation Scale-Cognitive; CDR, Clinical Dementia Rating; CG, Control Group; DSM-III, Diagnostic and Statistical Manual of Mental Disorders, Third Edition; DSM-IV, Diagnostic and Statistical Manual of Mental Disorders, Fourth Edition; IG, Intervention Group; MCI, Mild Cognitive Impairment; MMSE, Mini-Mental State Examination; MoCA, Montreal Cognitive Assessment; NR, not reported; SD, Standard Deviation; UK, The United Kingdom; USA, The United States of America; VD, Vascular Dementia.

#### 3.1.2 Geographic distribution and settings

The studies were conducted across Europe, North America, and Asia, with no representation from Africa or South America. Research settings included nursing homes, care facilities, memory clinics, community centers, neurology outpatient clinics, and participants' homes.

#### 3.1.3 Cognitive assessment tools

Standardized cognitive evaluations utilized the Mini-Mental State Examination (MMSE), Alzheimer's Disease Evaluation Scale-Cognitive (AES-C), Alzheimer's Disease Assessment Scale-Cognitive Subscale (ADAS-Cog), and Montreal Cognitive Assessment (MoCA). Scoring directions differ inversely between MMSE/MoCA and ADAS-Cog/AES-C. In addition, only one study relying on the identical dataset applied AES-C and ADAS-Cog, respectively. To reduce statistical heterogeneity and secure methodological rigor, the quantitative meta-analysis in the present study only incorporated studies adopting MMSE or MoCA as primary cognitive outcome measures. Studies utilizing ADAS-Cog, AES-C and other alternative scales, as well as those with unavailable data for pooled effect estimation, were subjected to supplementary qualitative analyses separately.

#### 3.1.4 Data overlap and follow-up

Four studies adopted two shared patient databases from Germany (Graessel et al., 2011; Luttenberger et al., 2012) and Denmark (Waldorff et al., 2012; Phung et al., 2013) to conduct serial follow-up analyses. Specifically, the studies by Graessel et al. (2011) and Luttenberger et al. (2012) drew on identical case datasets: Graessel et al. (2011) reported outcomes at baseline and immediately after intervention, while Luttenberger et al. (2012) extended observation and supplemented follow-up data collected at 10 months post-intervention based on the same cohort.

A comparable situation applies to the two Danish studies originating from the Danish Alzheimer Study (DAISY) cohort. Waldorff et al. (2012) compared outcomes between the intervention and control groups at baseline and immediately following the intervention, whereas Phung et al. (2013) conducted intergroup comparisons at baseline and the 24-month follow-up after intervention (Waldorff et al., 2012; Phung et al., 2013).

The post-intervention follow-up duration across included studies ranged from 1 week to 24 months. Among the 18 RCTs enrolled in this systematic review, only 8 implemented post-intervention follow-up assessments, and the remaining 10 contained no follow-up data.

### 3.2 Nurse-led cognitive interventions

#### 3.2.1 Intervention delivery and team composition

Table 3 presents a summary of the intervention characteristics. In all included studies, interventions were consistently delivered by nursing-led teams, comprising two distinct configurations: single-nurse teams and multidisciplinary teams. The nursing workforce represented a broad spectrum of specialties, including registered nurses, geriatric nurses, public health nurses, psychiatric nurses, and nurse care managers. Multidisciplinary teams, in addition to nursing staff, incorporated occupational therapists, social workers, psychologists, facility staff, and other allied health professionals.

**Table 3:** Characteristics of nurse-led non-pharmacological interventions.

No.	First author (year)	Intervention provider	Intervention characteristics						
			Type of cognitive intervention	Contents of intervention	Control group	Frequency and duration	Cumulative intervention duration	Time points for data collection	Main outcomes
1	Lai et al. (2004)	• Registered nurse, • Occupational therapist • Social worker	RT	Specific reminiscence to stimulate recall by discussing their life experiences and the events of past.	Usual care	Once a week for 30 min/6 weeks	180 min	• T0: Baseline • T1: Immediately post-intervention • T2: 6 weeks post-intervention	• Cognition • ADL • Dementia care mapping • The status of residents
2	Callahan et al. (2006)	Geriatric nurse practitioner	Behavioral intervention	Eight behavioral intervention protocol included personal care, repetitive behavior, mobility, sleep disturbances, depression, agitation and aggression, delusions or hallucinations and the caregiver's physical health.	Usual care	Be sustained/1 year	1 year	• T0: Baseline • T1: The 6th month of the intervention • T2: immediately post-intervention • T3: 6 months post-intervention	• Cognition • ADL • Neuropsychiatric • Depression in dementia
3	Tadaka and Kanagawa (2007)	• Public health nurse • Psychologist • Facility staff	RT	Reminiscence group program Included welcome meeting, health check and RT session. RT session was executed using themes and prompt suitable to the subjects' characteristics and life histories. The leader would attempt to keep the theme alive and to help subjects share memories with other subjects in the group.	Standard care	Once a week for 60–90 min/8 weeks	480–720 minutes	• T0: baseline • T1: immediately post-intervention • T2: 6 months post-intervention	• Cognition • ADL
4	Wang (2007)	• Psychiatric nurse • Geriatric nurse	RT	Reminiscence group therapy included different themes led by one intervenner with one co-intervenner. Themes included first meeting, childhood experiences etc. using memory triggers such as photographs, old time music etc.	Usual therapy	Once a week for 60 min/8 weeks	480 min	• T0: baseline • T1: immediately post-intervention	• Cognition • Depression
5	Chien and Chan (2009)	Nurse care manager	Psychological intervention	Nurse-led family psychoeducation programm formulated a structured education and supportive program on effective dementia care for each family. Family sessions consisted of education, sharing and discussion, psychological support, and problem solving.	Routine care	Once bi-weekly for 2 h/6 months	1440 min	• T0: baseline • T1: one week post-intervention • T2: 15 months post-intervention	• Cognition • Neuropsychiatric • Rate of institutionalization
6	Hsieh et al. (2010)	Geriatric psychiatric nurse	RT	Reminiscence group therapy encouraged participants to tell their stories and to gather old photos, albums, or meaningful materials to use when they shared their personal life experiences.	Usual care	Once a week for 40–50 min/12 weeks	480–600 min	• T0:baseline • T1: immediately post-intervention	• Neuropsychiatric • Depression • Apathy(behavior, cognition, emotion)
7	Graessel et al. (2011)	Registered nurse, Aide	• Motor stimulation • Practice in ADL • CS	• 10 min for introduction including greetings, a group song or a disscution • 30 min for motor exercises • 30 min for cognitive tasks, such as solving word jumbles • 40 min for ADL	Usual care	Six days per week for 2 h/12 months	>600 h	• T0: baseline • T1: immediately post-intervention	• Cognition • Depression • ADL
8	Luttenberger et al. (2012)	Registered nurse, Aide	• Motor stimulation • Practice in ADL • CS	• 10 min for introduction including greetings, a group song or a disscution • 30 min for motor exercises • 30 min for cognitive tasks, such as solving word jumbles • 40 min for ADL	Usual care	Six days per week for 2 h/12 months	>600 h	• T0: baseline • T1: immediately post-intervention • T2: 10 months post-intervention	• Cognition • Depression • ADL
9	Waldorff et al. (2012)	Experienced nurse	Psychological intervention	• Provide 7 individual counseling sessions • Provide outreach telephone counseling 5-8 times with 3–4 week intervals • Using log books • Experts to teach 5 standard courses to provide general and special information about dementia and support	Usual care	The 3rd, 6th, 12th month of the intervention/1 year	No reported	• T0: Baseline • T1: The 6th month of the intervention • T2: Immediately post-intervention	• Cognition • Depression • ADL • Neuropsychiatric • QoL
10	Phung et al. (2013)	Experienced nurse	Psychological intervention	• Provide 7 individual counseling sessions • Provide outreach telephone counseling 5-8 times with 3–4 week intervals • Using log books • Experts to teach 5 standard courses to provide general and special information about dementia and support	Usual care	The 3rd, 6th, 12th month of the intervention/One year	No reported	• T0: Baseline • T1: 24 months post-intervention	• Cognition • Depression • ADL • Neuropsychiatric • QoL
11	Van Bogaert et al. (2013)	Registered nurse	RT	Reminiscence therapy based on the SolCos model. Four standarded topics (family, profession, holiday, and games) was explored and four personalized memory boxes were provided to supplement the content in familar places.	Usual care	Twice a week for 45 min/4 weeks	360 min	• T0: baseline • T1: immediately post-intervention	• Cognition • Depression • Neuropsychiatric
12	Jha et al. (2013)	Nurse	Psychological intervention	Received a recovery-focused pre-diagnostic wellbeing assessment and counseling, diagnostic consultation with written feedback and post-diagnostic support.	Usual care	Once a month for 1 h/6 months	360 min	• T0: baseline • T1: immediately post-intervention	• Cognition • Depression • QoL • Wellbeing
13	Thyrian et al. (2017)	Nurse	Dementia care management	A model of collaborative care aims to provide optimal care by integrating multiprofessional and multimodel strategies.	Usual care	Once a month for 1 h/6 months	360 min	• T0: baseline • T1: 6 months post-intervention	• Cognition • Neuropsychiatric • QoL
14	Bademli et al. (2019)	Registered nurse	RT	Reminiscence group therapy allowed the participants to remember their important experiences, positive experiences, and achievements in the past, such as childhood experiences, fastivals, memorable traveled places, favorite foods, important historical terms, achievements, and music of the term.	Standard nursing	Once a week for 1 h/6 months	1440 min	• T0: Baseline • T1: immediately post-intervention	• Cognition • Depression • QoL
15	Lok et al. (2020)	Registered nurse	CS	Cognitive stimulation therapy based on Roy's adaptation model. The session titles included physical games, food, number games, team quiz, etc.	Standard nursing	Twice a week for 45 min/7 weeks	630 min	• T0: baseline • T1: immediately post-intervention	• Cognition • Coping • QoL
16	Yilmaz and Asiret (2021)	Registered nurse	Doll therapy	Life-like, lightweight dolls made of soft materials with life-like hair, in accordance with the guidelines on using dolls.	Standard nursing	Be sustained/8 weeks	8 weeks	• T0: baseline • T1: the 4th week of the intervention • T2: immediately post-intervention	• Cognition • Neuropsychiatric
17	Juniarti et al. (2021)	Registered nurse	• CS • Motor stimulation	• 30 min for reading activity • 30 min for the low-impact aerobic activity	Regular activity, but after the post-test, control group received the same treatment.	Three times a week for 60 min/4 weeks	720 min	• T0: baseline • T1: immediately post-intervention	• Cognition • Physical activity
18	Yan et al. (2024)	Nurse	CS	Staged intergral art-based cognitive intervention group. It included three stages: art experience, art enlightenment, and art exploration.	Regular activity, but after the post-test, control group received the same treatment.	24 sessions for 90 min per session/16 weeks	2160 min	• T0: baseline • T1: immediately post-intervention • T2: 6 months post-intervention	• Cognition • Depression • QoL • ADL

ADL, Activity of Daily Living; CS, Cognitive Stimulation; NR, Not Reported; QoL, Quality of Life; RT, Reminiscence Therapy.

#### 3.2.2 Types of cognitive interventions

RT was the most frequently employed intervention, utilized in six studies (Lai et al., 2004; Tadaka and Kanagawa, 2007; Wang, 2007; Hsieh et al., 2010; Van Bogaert et al., 2013; Bademli et al., 2019). Other modalities included: behavioral interventions (Callahan et al., 2006), psychological interventions (Chien and Chan, 2009; Waldorff et al., 2012; Jha et al., 2013; Phung et al., 2013), motor stimulation (Juniarti et al., 2021), ADL practice (Graessel et al., 2011; Luttenberger et al., 2012), cognitive stimulation (Lok et al., 2020; Yan et al., 2024), and doll therapy (Yilmaz and Asiret, 2021). These interventions were categorized into two models: single-model interventions (Lai et al., 2004; Callahan et al., 2006; Tadaka and Kanagawa, 2007; Wang, 2007; Chien and Chan, 2009; Hsieh et al., 2010; Yilmaz and Asiret, 2021; Yan et al., 2024) and multimodal/integrated models (Graessel et al., 2011; Juniarti et al., 2021). All control groups received either Usual Care or Standard Care.

#### 3.2.3 Intervention frequency and duration

A majority of studies implemented intermittent intervention schedules, administered weekly, biweekly, or monthly. In contrast, only two studies (Callahan et al., 2006; Yilmaz and Asiret, 2021) adopted daily, continuous intervention protocols. This resulted in substantial variability in CID, ranging from 180 min to 12 months.

#### 3.2.4 Outcome assessment timing

Most studies conducted immediate post-intervention evaluations without follow-up assessments. However, eight studies incorporated longitudinal follow-ups, with durations spanning 1 week to 24 months.

#### 3.2.5 Outcome variables

Outcomes associated with NLNPIs were multifaceted, encompassing: cognitive function, ADL, depressive symptoms, quality of life, neuropsychiatric symptoms, and overall wellbeing. A comprehensive summary of NLNPIs characteristics across the 18 studies is provided in Table 2.

### 3.3 Quality assessment

**Figure 2:**
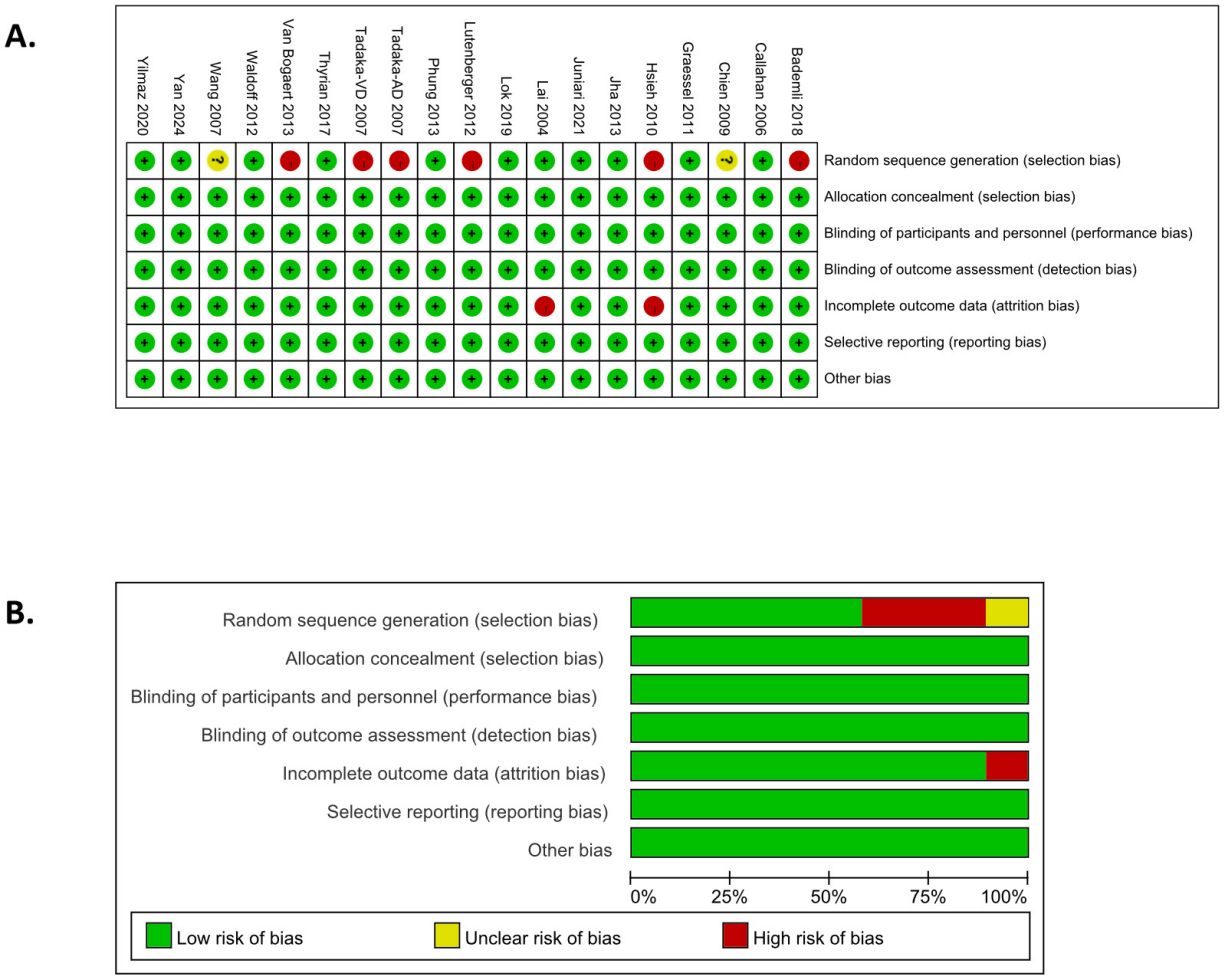
**(A)** Risk of bias graph in included studies; **(B)** Risk of bias summary in included studies.

**Figure 3:**
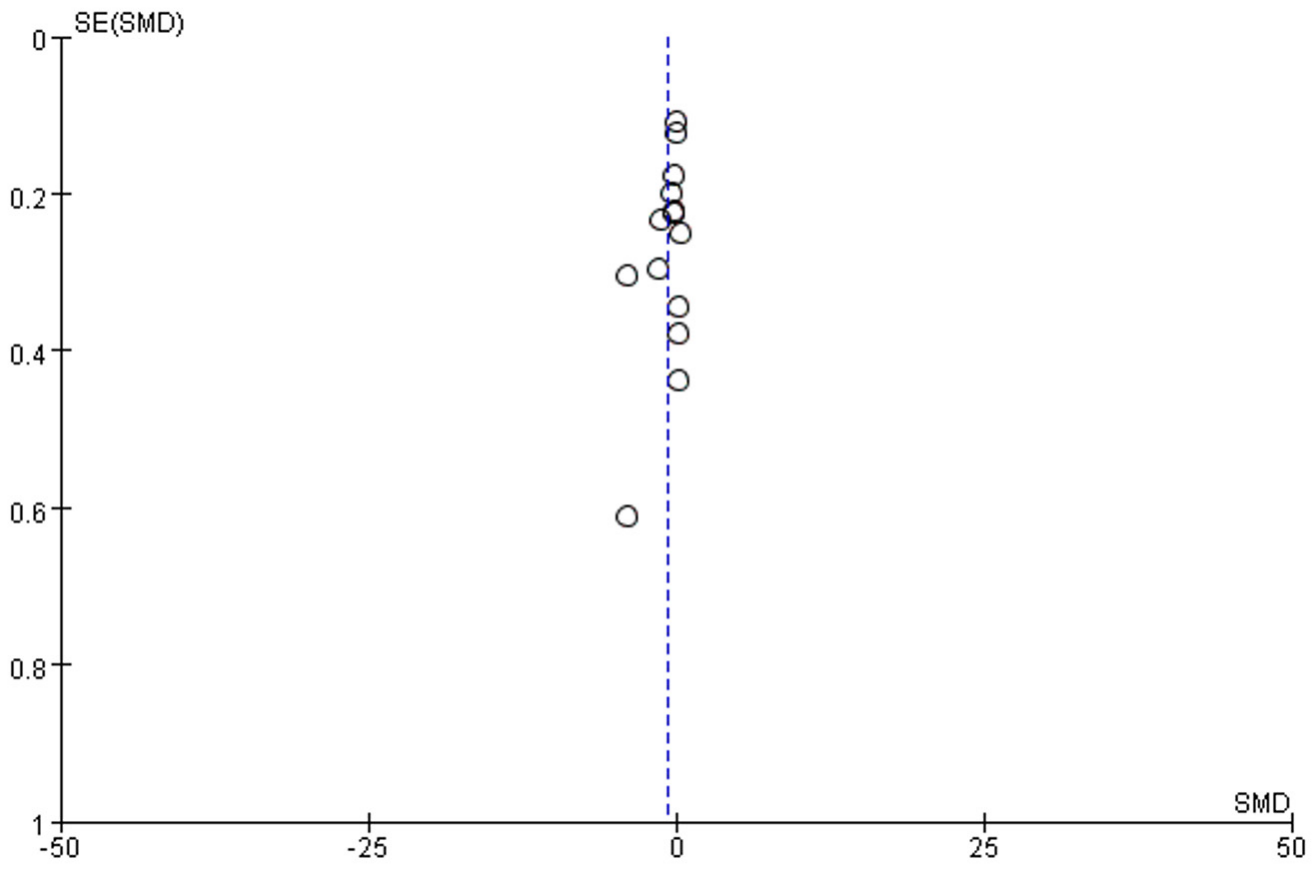
Funnel plot.

Figure 2A presents the risk of bias for 18 RCTs. Figure 2B shows a risk of bias summary for these studies. Five RCTs were identified as having a high risk of bias arising from the randomization process. The funnel plot conducted to evaluate the likelihood of publication bias was symmetric (Figure 3).

### 3.4 Efficacy of NLNPIs to improve cognition

**Figure 4:**
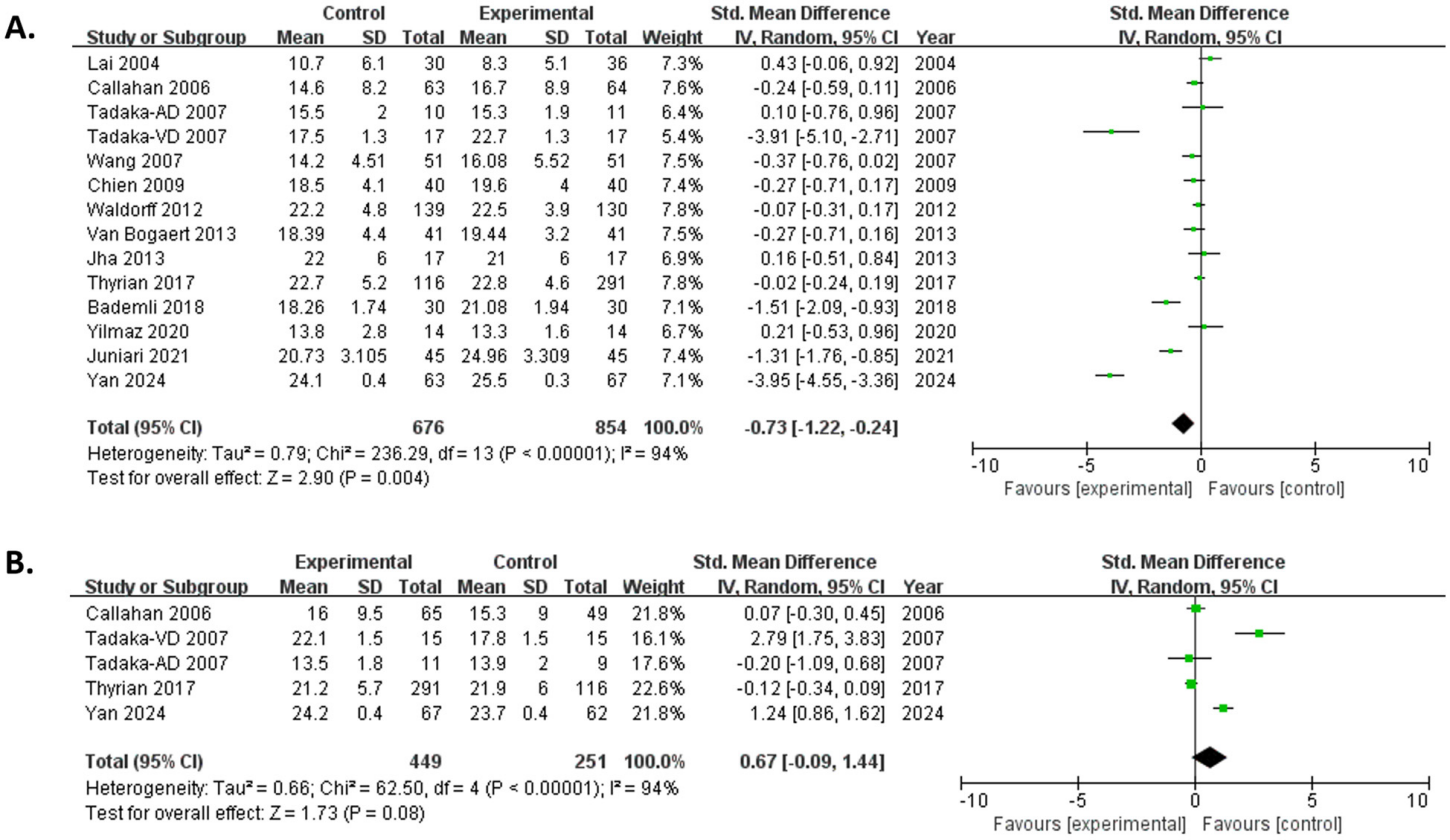
Forest plot of efficacy of NLNPIs to improve cognition. **(A)** Short-term effects (outcomes assessed ≤1 week post-intervention); **(B)** Long-term effects (outcomes assessed with a 6-month follow-up).

For short-term effects (outcomes assessed ≤ 1 week post-intervention), 13 studies met inclusion criteria. A random-effects model was applied given anticipated heterogeneity, revealing a statistically significant effect size (*d* = −0.73, 95% CI [−1.22, −0.24], *p* = 0.004). However, high heterogeneity (*I*^2^ = 94%) was observed, prompting evaluation via Q statistic (χ^2^ = 236.29) (Figure 4A).

Long-term effect assessment faced significant challenges due to excessive variability in follow-up durations (6 weeks to 24 months). To mitigate this, only four studies with 6-month follow-up assessments were included in the meta-analysis, as non-6-month time points lacked sufficient data (typically one study per time point), rendering robust pooling unfeasible.

The exclusion of non-6-month follow-ups was critical for maintaining analytical rigor, as insufficient study numbers at alternative time points would compromise statistical power and reliability. For the included 6-month studies, a random-effects model showed no statistically significant effect (*d* = 0.67, 95% CI [−0.09, 1.44], *p* = 0.08), with high heterogeneity (*I*^2^ = 94%) confirmed via Q statistic (χ^2^ = 62.50) (Figure 4B).

Although the following studies were excluded from the meta-analysis, we performed a supplementary analysis on them. Both studies by Graessel et al. (2011) and Luttenberger et al. (2012) adopted the ADAS-Cog as the evaluation instrument. Their findings demonstrated that a highly standardized, multicomponent group non-pharmacological intervention delivered in nursing homes could delay cognitive decline in patients with dementia for a minimum of 12 months, yet no long-term cognitive benefit was detected at the 22-month follow-up after intervention completion.

Hsieh et al. assessed the cognitive status of dementia patients using the AES-Cog scale, and their study indicated that mild-to-moderate dementia patients in the intervention group achieved specific improvements in the cognitive apathy score following 12 intervention sessions (Hsieh et al., 2010).

These outcomes further validate the cognitive-improving effect of NLNPIs for dementia patients. Lok et al. (2020) employed the MMSE for cognitive assessment, but their study was excluded from the meta-analysis owing to unavailable standard deviation data (Lok et al., 2020). Nevertheless, this study still revealed that the experimental group exhibited statistically significantly superior cognitive function compared with the control group at the 7th-week endpoint assessment.

In summary, the results of this supplementary analysis are consistent with the primary analysis outcomes of the present study in terms of effect direction and statistical significance.

### 3.5 Subgroup analysis

**Figure 5:**
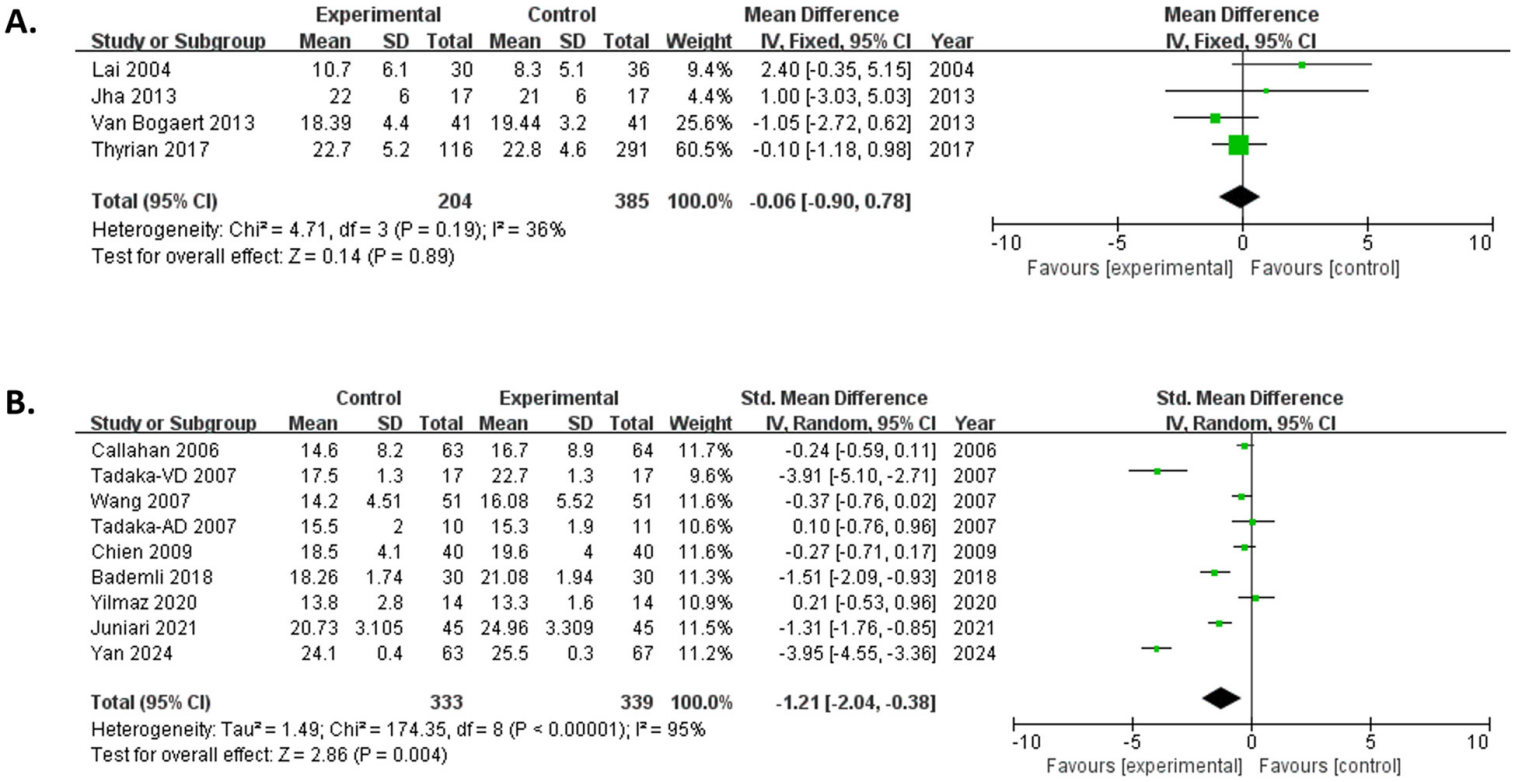
Forest plot of efficacy of subgroup analysis. **(A)** Low-dose effects; **(B)** High-dose effects.

Within the dataset of the present study, a CID of 480 min served as the optimal stratification cut-off point that effectively discriminated between effective and ineffective interventions. Dose strata were divided using this threshold to explore the impacts of interventions at different dose levels on short-term cognitive function:

Low-dose group (CID < 480 min): The intervention effect failed to reach statistical significance (*d* = −0.06, 95%CI [−0.90, 0.78], *p* = 0.89), with moderate between-study heterogeneity (*I*^2^ = 36%) (Figure 5A).

High-dose group (CID ≥ 480 min): The intervention yielded a statistically significant beneficial effect (*d* = −1.21, 95%CI [−2.04, −0.38], *p* = 0.004), accompanied by substantial between-study heterogeneity (*I*^2^ = 95%) (Figure 5B).

## 4 Discussion

Pharmacological treatments for dementia have demonstrated limited efficacy in improving cognitive function and remain controversial due to safety concerns and variable patient responses (Kurkinen, 2023). In contrast, NPIs have gained traction owing to their cost-effectiveness, minimal adverse effects, and broader applicability across dementia subtypes (Eaglestone et al., 2023). Prior research highlights the multidisciplinary nature of NPIs delivery, with nurses, physicians, social workers, physiotherapists, and psychotherapists collaborating as individual practitioners or within integrated care teams (Burnand et al., 2024). However, emerging evidence underscores the pivotal role of nurses as primary coordinators and leaders of NPIs, leveraging their expertise in patient-centered care to optimize intervention adherence and outcomes (Suh et al., 2023).

Compared with previous systematic reviews, the present study features a higher level of evidence, a larger sample size, and updated research data. Furthermore, it incorporates an exploratory dose-response analysis, which is expected to provide more reliable evidence-based references for clinical dementia care.

The meta-analysis results confirm that NLNPIs exert a clinically significant therapeutic effect on cognitive function in dementia patients, aligning with findings from prior studies. The core reasons why nurses serve as the optimal implementers of NPIs are summarized as follows. First, nurses provide full-cycle care for individuals with dementia throughout hospitalization, home-based care, and long-term institutional follow-up. Their frequent and continuous contact with patients and family caregivers enables real-time monitoring of dynamic changes in cognitive function and neuropsychiatric symptoms, supporting individualized and flexible adjustment of NPI protocols. Such high-intensity longitudinal follow-up cannot be achieved by specialized professionals, such as physicians and rehabilitation therapists (Gauthier et al., 2022).

Second, licensed clinical nurses receive standardized practical training focusing on NPIs for cognitive impairment. They are proficient in the standard operating procedures of mainstream NPI modalities, including reminiscence therapy, cognitive stimulation therapy, and reality orientation therapy. This guarantees standardized intervention implementation and minimizes operational bias.

Third, nurses can simultaneously deliver health education and psychological counseling for family caregivers, effectively improving patients' intervention engagement and resolving the critical problem of high dropout rates that hinders the large-scale application of NPIs (Chao et al., 2020).

Fourth, nurses are capable of delivering NPIs across diverse care settings, including communities, long-term care facilities, and patients' homes, which avoids the need for patients to attend specialized outpatient services. This delivery model is particularly suitable for patients with moderate to severe dementia with limited mobility (Bergman et al., 2013).

Therefore, the professional leadership of nurses constitutes the cornerstone for ensuring the efficacy of NPIs in improving cognitive function among dementia patients, while also driving standardized, evidence-based development of dementia cognitive care. Consequently, we strongly advocate for elevating nurse participation in policy and guideline formulation for dementia patient management to optimize intervention outcomes.

A two-decade review of NPIs targeting cognitive enhancement in dementia reveals RT as the most widely adopted nurse-led intervention, with six studies employing this approach. RT, a well-established non-pharmacological cognitive intervention, demonstrates significant clinical utility in dementia treatment, supported by robust empirical evidence (Tominari et al., 2021; Yanagida et al., 2024a).

Dementia patients frequently experience progressive cognitive decline, emotional dysregulation, and a loss of self-identity, all of which RT addresses by stimulating recollection of past memories and life narratives. This therapeutic mechanism activates latent synaptic connections, enhancing neural plasticity and delaying deterioration in memory, orientation, and executive function (Cammisuli et al., 2022). RT is particularly efficacious for patients with mild-to-moderate dementia, yielding dual benefits: beyond cognitive improvement, it alleviates psychological distress, reduces agitation, withdrawal, and other behavioral and psychological symptoms, improves emotional wellbeing and subjective quality of life, and enhances daily living participation (Yanagida et al., 2024b).

In addition, with advancements in medical technology, digitally integrated RT in nurse-led dementia care not only achieves cognitive improvement outcomes comparable to traditional RT but also significantly reduces depression levels among patients (Moon and Park, 2020). As primary implementers, nurses tailor RT interventions—delivered individually or in groups—based on patients' cognitive capacities and life histories, solidifying its role as an indispensable non-pharmacological modality in dementia management.

Beyond classical RT, nurses increasingly employ innovative NPIs grounded in advanced theoretical frameworks, including motor stimulation (Juniarti et al., 2021), doll therapy (Henderson et al., 2024), pet companion therapy (Perkins et al., 2008; Zhang et al., 2024), and art therapy (Yan et al., 2024). Concurrently, digital technologies such as computer-assisted cognitive training (Hill et al., 2017), virtual reality interventions (Kim et al., 2019), and pet robotics (Petersen et al., 2017) are being widely adopted to enhance cognitive function. The integration of artificial intelligence, smart devices, and robotics into dementia care is anticipated to accelerate, necessitating that nurses proactively leverage digital tools to optimize therapeutic efficacy (Su et al., 2022; Mekulu et al., 2025).

Nevertheless, NLNPIs do not consistently yield beneficial outcomes, and several investigations have failed to detect significant cognitive improvements following these interventions. For instance, Yilmaz's research observed no meaningful cognitive benefits of doll therapy among individuals living with dementia (Yilmaz and Asiret, 2021).

Furthermore, although NPIs are recommended as first-line strategies for managing behavioral and psychological symptoms of dementia in patients with AD, their efficacy diminishes substantially or disappears entirely in advanced dementia stages. Patients in the late phase suffer from severe cognitive impairment, impaired communication, reduced mobility and heavy reliance on care providers, making it impossible for them to fully cooperate with the intervention procedures. We explicitly emphasize that such NPIs cannot replace pharmacotherapy and palliative care for severe dementia patients (Profyri et al., 2022).

In addition, for certain mixed dementia subtypes such as AD combined with vascular dementia, simultaneous management of both Alzheimer's pathology and cerebrovascular lesions takes clinical priority. Apart from standard anti-dementia medications for AD, lipid-lowering agents and antiplatelet drugs are also required for comprehensive disease control (Wu and Fuh, 2025).

This study verifies that NLNPIs can improve cognitive function in patients with dementia, and such benefits persist at least until the completion of intervention. Nevertheless, 6 months after intervention termination, patients' cognitive performance largely declined back to the pre-intervention baseline level. This finding aligns with Luttenberger et al. (2012) longitudinal trial involving 61 patients with primary degenerative dementia across five German nursing homes. Their MAKS therapy—a standardized protocol combining motor stimulation, ADL practice, and cognitive stimulation—demonstrated long-term efficacy only for ADL, with no sustained cognitive benefits observed 10 months post-treatment (Luttenberger et al., 2012).

The transient nature of NLNPIs effects probably stems from multiple factors. First, irreversible neurodegenerative pathology in dementia patients undermines long-term compliance, particularly as disease progression exacerbates cognitive decline (Özişli and Kara, 2024; Wu et al., 2024). Additionally, inadequate family caregiving support exacerbates this issue, as highlighted by studies showing reduced sustainability of nurse-led interventions in such contexts (Schulz and Martire, 2004). Third, systemic barriers, including a lack of standardized intervention protocols and fragmented multidisciplinary collaboration, further hinder long-term efficacy. Future research must prioritize disease-stage-specific standardized protocols, alternative care plans for patients lacking family support, and sustained multidisciplinary teamwork, with nurses playing a pivotal role in these frameworks.

Current evidence supports the positive impact of NLNPIs on dementia cognition. However, few studies propose actionable, evidence-based programs, and dose-response relationships remain contentious. The World Health Organization recommends ≥150 min of moderate cardiorespiratory exercise or 75 min of high cardiorespiratory exercise weekly, paired with resistance exercise, to mitigate cognitive decline in older adults (Cayon, 2016). Liu et al. (2024) further refined these guidelines, identifying age-specific optimal dosages for computer-assisted cognitive training: 25–30 min/day, 6 days/week for participants under 60, and 50–55 min/day, 6 days/week for those aged ≥60, underscoring the dose-dependent nature of cognitive improvement (Liu et al., 2024).

We defined the CID as the intervention dose, which is jointly determined by three components: single-session duration, weekly intervention frequency, and total number of intervention weeks. Nevertheless, the optimal combination of these three parameters has not yet been clarified. Our analyses suggest that a CID of 480 min may serve as the critical threshold to generate cognitive benefits, whereas the observed dose-dependent trend should be interpreted with caution owing to the limited number of included studies for dose-response analysis, potential aggregation bias derived from summary data, and the *post-hoc* identification of this threshold. The 480-min threshold is merely an exploratory finding rather than a definitive conclusion, which requires further verification by prospective studies adopting individual participant-level raw data in the future.

This threshold effect may be jointly determined by the pathological characteristics of dementia, the activation patterns of cerebral neural plasticity, and the consolidation mechanisms of cognitive learning (Chin, 2023).

Dementia's core pathologies—neuronal apoptosis, synaptic loss, impaired hippocampal/prefrontal plasticity, and cholinergic deficits—are irreversible and progressive, limiting long-term intervention efficacy (Ng et al., 2019; Kim et al., 2022). Furthermore, NLNPIs counteract these deficits by stimulating residual neural cells, inducing synaptogenesis, and reconstructing damaged circuits. However, a threshold effect exists in synaptic remodeling and neural plasticity initiation, while cognitive relearning adheres to the “law of exercise and reinforcement,” requiring sufficient stimulation intensity and repetition to consolidate new pathways (Wang et al., 2025). Low-dose interventions fail to meet these thresholds, resulting in suboptimal outcomes (Belleville et al., 2011). Future studies are recommended to prioritize intervention duration as a core research direction, so as to provide robust evidence for clinical nursing practice.

Marked clinical heterogeneity across the included studies in the present research is primarily attributable to disparities in dementia severity and intervention settings, both of which may substantially modulate our pooled findings.

With regard to heterogeneity stemming from dementia severity, the lack of consistent criteria for disease staging across primary studies constitutes the fundamental cause. To date, no unified consensus has been established in academia to stratify mild, moderate and severe dementia. Although the diagnostic criteria, such as MMSE, Clinical Dementia Rating scale, and Diagnostic and Statistical Manual of Mental Disorders, are widely adopted to confirm dementia diagnosis, individual studies adopt distinct cut-off values for severity stratification without standardized reference benchmarks. Furthermore, most eligible studies only report the overall baseline cognitive status of participants, without detailed stratification and statistical analyses according to dementia severity. A subset of trials additionally enrolled individuals with mild cognitive impairment or subjective cognitive decline who have not progressed to overt dementia, further exacerbating heterogeneity in disease severity within the pooled sample.

In terms of heterogeneity related to intervention delivery settings, the included studies fall into two major categories: institutional care and community- or home-based care, covering nursing homes, professional care centers, memory clinics, neurology outpatient departments, and home family care services. Patients recruited from different settings differ significantly in ADL, cognitive reserve, degree of functional impairment, and accessible care resources. Recipients receiving institutional care are mostly diagnosed with moderate to severe dementia accompanied by more severe physical and cognitive deficits, and can receive long-term, standardized professional nursing interventions. By contrast, participants under community and home care predominantly present with mild dementia and retain partial independent living capacity, relying largely on informal family caregivers for intervention implementation.

Essential discrepancies in care intensity, intervention frequency and professional guidance across diverse settings directly alter the actual delivery efficacy of interventions. This introduces considerable clinical heterogeneity into the overall pooled analyses and undermines the stability and reliability of the dose–effect relationship.

Apart from the above factors, other clinical characteristics, including dementia subtypes, intervention characteristics and follow-up duration, also contribute to heterogeneity. This finding highlights the urgent necessity of conducting large-sample, rigorously designed RCTs with standardized intervention protocols and rigorous blinding design in future research.

## 5 Limitation

This study had the following limitations: First, substantial heterogeneity was detected across the included studies, and there were insufficient data to perform subgroup analyses stratified by potential sources of heterogeneity. In addition, the number of high-quality studies included was limited. Additional high-quality trials are warranted to verify and refine our findings and strengthen the reliability and validity of the results. Second, the threshold identified in the dose-response analysis was derived from exploratory rather than confirmatory analyses. Large-scale prospective cohort studies or individual participant data meta-analyses are still required before this threshold can be adopted as a clinical reference. Third, limiting the literature search to English publications may lead to language bias.

## 6 Conclusion

Compared with routine nursing care, NLNPIs can improve cognitive function in patients with dementia, and the therapeutic benefits last at least until the termination of interventions. Exploratory analyses suggest that the intervention effect may have a dose-dependent relationship with total intervention duration; however, its stability and generalizability need to be evaluated cautiously. Based on currently available study data, the long-term efficacy of such interventions remains unclear. It is recommended to conduct large-sample, high-quality RCTs with standardized intervention protocols and rigorous blinding designs, to thoroughly verify the long-term efficacy and dose thresholds and provide more reliable evidence-based foundations for clinical dementia care.

## Data Availability

The original contributions presented in the study are included in the article/Supplementary material, further inquiries can be directed to the corresponding authors.
