# Supplementary material for: Nurse-led non-pharmacological interventions for cognitive improvement in dementia patients: a systematic review and meta-analysis of randomized controlled trials
Source: Front Aging Neurosci. 2026 Jul 24;18:1876966. doi: 10.3389/fnagi.2026.1876966 (PMC13447354; doi:10.3389/fnagi.2026.1876966)
Supplement: Supplementary file 2 [file Data_Sheet_2.pdf]

Details of MMSE or MoCA in included studies

Time points for data collection	control group [post-test(T1/T2/T3)]			exprimental group [post-test(T1)]			exprimental group [post-test(T2)]			exprimental group [post-test(T3)]		
First author (Year)	Mean	SD	n.	Mean	SD	n.	Mean	SD	n.	Mean	SD	n.
Lai(2004)	10.7	6.1	30	8.3	5.1	36						
	10.5	6.1	27				8.4	5	30			
Callahan(2006)	16	7.1	62	16.9	8.8	72						
	14.6	8.2	63				16.7	8.9	64			
	15.3	9	49							16	9.5	65
Tadaka（2007）-AD	15.5	2	10	15.3	1.9	11						
	13.9	2	9				13.5	1.8	11			
Tadaka（2007）-VD	17.5	1.3	17	22.7	1.3	17						
	17.8	1.5	15				22.1	1.5	15			
Wang (2007)	14.2	4.51	51	16.08	5.52	51						
Chien (2009)	18.5	4.1	40	19.6	4	40						
	19.1	4.8	40				19.8	5.8	40			
Hsieh (2010)	NR											
Graessel (2011)/ Lutenberger (2012)	NR											
Waldoff (2012)/ Phung (2013)	23	3.6	149	23	3.8	144						
	22.2	4.8	139				22.5	3.9	130			
	17.9	7.1	94							17.8	6.7	84
Van Bogaert (2013)	18.39	4.4	41	19.44	3.2	41						
Jha (2013)	22	6	17	21	6	17						
Thyrian (2017)	21.9	6	116	21.2	5.7	291						
Bademli (2018)	18.26	1.74	30	21.08	1.94	30						
Lok (2019)	14.5	NR	30	20	NR	30						
Yilmaz (2020)	13.2	2	14	11.9	2.7	15						
	13.8	2.8	14				13.3	1.6	14			
Juniari (2021)	20.73	3.105	45	24.96	3.309	45						
Yan (2024)	24.1	0.4	63	25.5	0.3	67						
	23.7	0.4	62				24.2	0.4	67			

Note : T1, T2, and T3 denote different time points for data collection across various studies. For specific details, please refer to Table 3 "Characteristics of nurse-led non-pharmacological interventions".
Abbreviations: MMSE, Mini-Mental State Examination; MoCA, Montreal Cognitive Assessment; NR, No Reported; SD, Standard deviation;
